# PRICE: A Personalized Recursive Intelligent Cost Estimation Framework for Rare Disease Diagnosis

**DOI:** 10.21203/rs.3.rs-6623705/v1

**Published:** 2025-06-12

**Authors:** Mengshu Nie, Yujing Yao, Junyoung Kim, Cong Liu

**Affiliations:** 1 Boston Children’s Hospital, Department of Pediatrics, Division of Genetics and Genomics, Boston, MA; 2 Gertrude H. Sergievsky Center, Columbia University Irving Medical Center, New York, NY; 3 Children’s Hospital Informatics Program, Boston, MA

**Keywords:** Diagnostic workflow modeling, Cost-effectiveness analysis, AI-delegation mode

## Abstract

**Background:**

Rare disease diagnosis often involves complex procedures that can be both costly and time-consuming. Traditional cost-effectiveness analyses typically employ static models, applying uniform diagnostic strategies across diverse patient populations. With advancements in artificial intelligence (AI) and a growing emphasis on personalized medicine, there is a pressing need for dynamic frameworks that assess diagnostic cost-effectiveness at the individual patient level.

**Methods:**

We introduce the PRICE framework—a novel, tree-based analytical model designed to evaluate the cost-effectiveness of diagnostic strategies, accommodating both expert-alone and AI-delegation decision-making modes. The model computes the expected cost of a diagnostic process via a back-propagation algorithm and quantifies effectiveness through a utility-based approach. Parameters such as disease prevalence, test costs, test performance metrics, and turnaround time are incorporated, allowing for individualized assessments.

**Results:**

We conducted a case study applying the framework to four diagnostic strategies for developmental delay (DD) and multiple congenital anomalies (MCA). The results demonstrate how PRICE can support patient decision-making by modeling outcomes under varying parameters such as test cost and accuracy. Additionally, we show that changes in AI performance influence the selection of optimal cost-efficient strategies under AI delegation. To facilitate interpretation and engagement, we developed an interactive web-based tool that visualizes and simulates diagnostic pathways in real time, enhancing decision-making support for both clinicians and patients.

**Conclusion:**

PRICE is a flexible cost-effective analysis framework that captures the sequential and recursive nature of real-world diagnostic workflows, with the ability to be extended to future AI-integrated clinical practice. It enables personalized evaluations of diagnostic strategies from both economic and clinical perspectives, promoting more informed and individualized decision-making, especially in rare disease diagnosis.

## 1. Background

The modern diagnostic process, which begins once a patient encounters a health issue and seeks medical attention, is an iterative cycle of information gathering, interpretation and the selection of diagnostic strategies. For individuals with rare genetic disorders, diagnostic testing often follows a multitiered approach, with each tier progressively refining the diagnostic hypothesis [1]. The process usually starts with an initial screening to gather preliminary clinical information, followed by more targeted tests to gain deeper insights into the patient’s condition. Further tiers of testing may be conducted to reconfirm the diagnosis or explore alternative possibilities if the previous tests fail to yield a definitive diagnosis [2]. How to select the appropriate tests and determine their sequence is a critical aspect of diagnostic decision-making, as it directly influences the accuracy and efficiency of reaching a diagnosis [3, 4].

Schaafsma et al. [5] outlined four aspects to evaluate a diagnostic test: test characteristics, added value of the test, clinical outcome and cost-effectiveness. Newman-Toker et al. [6] emphasized the critical role of economic evaluation in enhancing diagnostic quality, particularly by minimizing the overuse or underuse of tests. However, current guidelines for cost-effectiveness analysis are often designed from the healthcare provider or societal perspective, overlooking patient-specific circumstances and preferences, which leads to generalized analyses that may not align with individual patient welfare [7]. For example, Li et al. [8] evaluated multiple genetic testing strategies for developmental delays (DD) and multiple congenital anomalies (MCA) and compared genome-wide and exome sequencing (GS and ES) with standard chromosomal microarray analysis (CMA) and gene panel tests. These comparisons assume a one-size-fits-all application of strategies across all patients if implemented in a clinical institution. However, with a growing focus on personalized medicine, especially with the advancement of utilizing artificial intelligence (AI) to tailor the diagnosis and treatment plans for individuals [9, 10], there is an increasing demand for diagnostic strategies that adapt to individual patient characteristics [11]. This shift necessitates analytical frameworks capable of tailoring cost-effectiveness analysis to the individual level, guiding test selection not based on population averages but rather on the expected value for a specific patient.

Achieving such a personalized diagnosis requires information from multiple sources, such as the pretest probability of disease, the yielding rate of a given test, the patient’s phenotypes, demographics and clinical history—often within a probabilistic reasoning process [12]. The manual process of collecting these data points and conducting probabilistic reasoning can be cognitively burdensome and error- prone, which underscores the need for intelligent systems that can support or automate diagnostic reasoning [13]. Advances in artificial intelligence (AI) provide promising solutions: AI can rapidly analyze large-scale patient data, predict diagnostic yield based on individual profiles, and facilitate efficient diagnostic decision-making. Furthermore, the human-AI collaboration mode has shown substantial potential in augmenting healthcare professionals’ cognitive strength, leveraging AI’s analytical capabilities while preserving expert oversight and final judgment [14, 15]. Building on the significance of patient-centered diagnostic testing procedures and the growing potential of human-AI collaboration, our study presents a novel tree-based analytical framework that enables individualized cost-effectiveness analysis of diagnostic strategies.

## 2. Methodology

### 2.1 Conceptualized Framework

**Figure 1.**
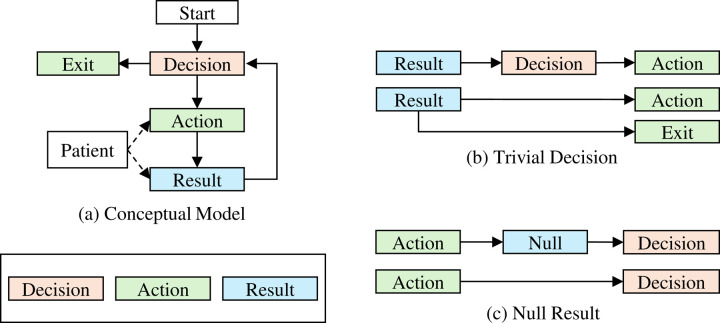
The Diagnostic Process a) A general diagnostic process follows an iterative *decision-action-result* sequence. For example, a patient with developmental delay needs to decide between two genetic tests (decision node), gene panel or exome sequencing (action nodes). After taking the test and obtaining the test result (result node), the patient may need to decide for further actions or exit the process (*exit* node) if no more action is needed. Note that the exit node can be considered a special type of action; however, we distinguish it explicitly to indicate that it represents the termination point of the diagnostic process. b) A decision node can be neglected if there is only one action node following (e.g., only one test to be considered) or when the patient exits the process. c) A new decision node can directly follow an action node if the action does not yield any diagnostic information.

We define a tree $T=\left(N,E\right)$ to represent a diagnostic process where $N=\left\{n_{i}|i=0,1,2,\ldots ,m-1\right\}$ denotes the set of m nodes (root node $n_{0}$) and where $E=\left\{e_{ij}|n_{j}\in children\left(n_{i}\right)\right\}$ denotes the set of directed edges connecting each parent node to its child nodes. The set of leaf nodes is defined as $\left\{n_{l}\in N|children\left(n_{l}\right)=\emptyset \right\}$. Figure 1a illustrates the generalized conceptual model of a diagnostic process. To capture the logic and structure of a real-world diagnostic workflow, we define three types of nodes in this diagnostic tree: *decision*, *action* and *result*. Each node may have various associated attributes, such as cost and turnaround time. A decision node $\left(D\right)$ acts as a clinical judgment or planning point at which the next clinical action will be selected among multiple options based on available information, such as the patient’s phenotypes or the prior test results. It is said to be *trivial* when followed by a single action node (Figure 1b). An action node $\left(A\right)$ corresponds to the execution of a diagnostic step, such as taking a specific test, attending a genetic consultation, or simply exiting the process. In certain cases, an action node may transition directly to a new decision node if it does not yield any diagnostic information (e.g., a genetic consultation; Figure 1c). A result node $\left(Y\right)$ takes either positive $\left(1\right)$ or negative $\left(0\right)$ values for a dichotomous diagnostic test or continuous numeric values for a quantitative test. These nodes collectively define the sequential and recursive nature of a diagnostic pathway. The process begins with the patient entering an initial decision node, which leads to one or more action nodes, such as the selection of a diagnostic test. Upon the completion of the action, the subsequent result node is directed to another decision node, whether to terminate the diagnostic procedure or proceed with the next action. The patient may exit the process if a conclusive diagnosis is reached at any stage or if all potential options have been exhausted; otherwise, the pathway continues recursively through additional *decision–action–result* cycles until an *exit* node is reached.

One of the key properties in defining this graph is the transition probability $P_{ij}$ from a parent node $n_{i}$ to its child node $n_{j}$ conditioning on the patient. The sum of the transition probabilities from any parent node to its children is equal to one (i.e., $\sum_{n_{j}\in children\left(n_{i}\right)}P_{ij}=1$). A transition probability is said to be trivial if a parent node is followed by a single child node (i.e., $P_{ij}=1$). The transition from results to decisions is always trivial by definition. Nontrivial transition probabilities include (1) from decision to action and (2) from action to result. The former represents the likelihood of selecting a particular diagnostic action. In clinical settings where standard protocols are followed [16], this probability is dichotomized, taking values of either 0 or 1. However, in practice, clinicians may deviate from standardized pathways [17], allowing these probabilities to be estimated empirically from historical test selection frequencies. Transition probabilities from action to result typically correspond to test performance and disease prevalence metrics, such as the diagnostic yield of a test, estimated as the percentage of positive results out of *all individuals* tested [18]. These metrics can be obtained from clinical studies or institutional data sources. Importantly, these probabilities are generally population-based and rarely account for individual patient variability. In AI-assisted approaches, the estimation of the transition probabilities from action to result is often derived by conditioning on patient-specific information (see Section 2.4).

### 2.2 Algorithm for Expected Cost Computing

To calculate the accumulated expected cost at each node $n_{i}$, denoted as $E\left({C^{+}}_{i}\right)$, we initialize each node with a base cost $C_{i}$, reflecting the direct cost associated with the node. Decision nodes include costs related to the expert time required to select the appropriate test; in practice, such decision-making costs are often related to clinical office visits. Action nodes are associated with the costs of diagnostic tests or clinical consultations, whereas result nodes, which indicate diagnostic outcomes, are usually associated with a zero cost. The cumulative expected cost at node $n_{i}$ can therefore be recursively defined as:

Equation 1. $$E\left(C_{i}^{+}\right)=\left\{\begin{array}{ll} \;\;\;\;\;\;\;\;\;\;\;\;\;\;\;\;\;\;\;\;\;\;\;\;\;\;\;\;\;\;\;\;\;\;\;C_{i}, & if\;children\left(n_{i}\right)\;=\;\emptyset \\ C_{i}+\sum_{n_{j}\in children\left(n_{i}\right)}P_{ij}*E\left(C_{j}^{+}\right), & otherwise \end{array}\right..$$

The primary objective is to compute the cumulative cost at the root node, which reflects the expected total cost of the entire diagnostic process across different potential pathways.

Building upon the tree structure defined in Section 2.1, a recursive algorithm is developed below to compute the expected cost of a diagnostic procedure, which is conceptually analogous to the backpropagation calculation [19]. Specifically, the computation begins at the leaf nodes which is analogous to the output layer in a neural network, and proceeds recursively upward through the tree to the root node, similar to propagating gradients backward from the output to the input layers. At each step, the expected cost at a given node is updated on the basis of the base costs and transition probabilities associated with its child nodes. This recursive nature ensures that diagnostic decisions and outcomes at the deeper layer of the tree are appropriately reflected in the cost estimation at earlier stages of the diagnostic process.


$$\bar{\underline{\begin{array}{l} \underline{Backpropagation\;Expected\;Cost\;Calculation\;\left(BECC\right).}\\ \text{Initialize}\;\text{the}\;\text{costs}\;\text{associated}\;\text{with}\;\text{all}\;\text{the}\;\text{nodes}\;(C_{i}).\\ \text{Initialize}\;\text{the}\;\text{probability}\;\text{associated}\;\text{with}\;\text{each}\;\text{edge}\;(P_{ij}).\\ expected\_cost=function\left(n_{i}\right)\{\\ \;\;\;\;\;\;\;\;\;\;if\;children\left(n_{i}\right)=\emptyset :\\ \;\;\;\;\;\;\;\;\;\;\;\;\;\;\;\;\;\;\;\;return\;C_{i}\\ \;\;\;\;\;\;\;\;\;\;expected=0\\ \;\;\;\;\;\;\;\;\;\;for\;n_{j}\;in\;children\left(n_{i}\right):\\ \;\;\;\;\;\;\;\;\;\;\;\;\;\;\;\;\;\;\;\;expected+=P_{ij}*expected\_cost\left(n_{j}\right)\\ \;\;\;\;\;\;\;\;\;\;expected+=C_{i}\\ \;\;\;\;\;\;\;\;\;\;return\;expected\\ \} \end{array}}}\;total\_cost=expected\_cost\left(n_{0}\right)$$

### 2.3 Cost Effectiveness Analysis

To complement the cost-estimation algorithm detailed in Section 2.2, we extend this framework to encompass diagnostic effectiveness and constitute a cost-effectiveness analysis (CEA) framework. CEA is a fundamental tool for evaluating healthcare interventions in relation to their associated costs, providing essential guidance for stakeholders in allocating resources efficiently and maximizing patient welfare [20]. To synthesize both cost and clinical benefit into a unified metric, we define the effective cost as the ratio of the expected diagnostic cost to the expected effectiveness [21]. This measure enables a nuanced evaluation of diagnostic pathways, balancing the trade-offs between economic burden and clinical effectiveness and facilitating more informed and personalized decision-making.

We first introduce the concept of an *outcome state* that characterizes the outcome of a patient’s diagnostic process with defined probabilities at an exit node. An *outcome state* is defined by three components: (a) the final result obtained at the exit and (b) the cumulative turnaround time TAT required to reach the exit. Formally, the outcome state at a leaf node i (i.e., an *exit* ending point of the process) is expressed as $S_{i}=\left(O_{i},TAT_{i}\right)$, where $O_{i}$ indicates the value of the final result node prior to exit and where $TAT_{i}=\sum_{j\in ancestors\left(i\right)}T_{j}$ indicates the total turnaround time accumulated across individual nodes j along the pathway to the exit. The overall effectiveness of the outcome state $S_{i}$ is quantified as

$$U\left(S_{i}\right)=\alpha *EGU_{i}+\left(1-\alpha \right)*e^{-\lambda TAT_{i}\_},$$

where $EGU_{i}$ represents the *expected gain in utility* (EGU) [22, 23] associated with the final outcome and its accuracy, whereas the second term penalizes longer diagnostic delays with an exponential decay function. If a diagnostic process may result in multiple possible outcomes, then its expected effectiveness is computed as the probability-weighted sum of the utilities across all possible outcome states. The weighting parameters, α, reflect the relative importance of test accuracy and time efficiency from the patient’s perspective. The decay-rate parameter λ controls the sensitivity of utility to the TAT, with a higher value of λ indicating greater urgency for timely diagnosis.

### 2.4 Extending the Framework to the AI-delegation Mode

**Figure 2.**
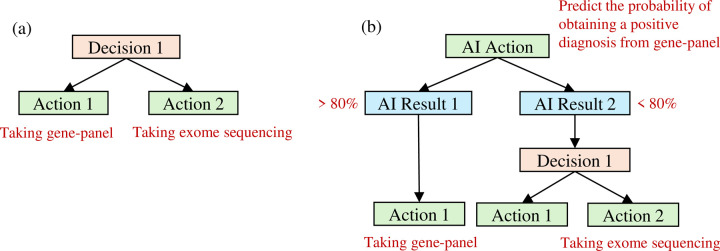
AI-delegation Mode Structure a) Expert-alone decision making. The physician directly makes a choice between two actions (e.g., gene panel vs. exome sequencing). b) AI-delegated decision making. Suppose that the AI action node at the top predicts how likely it is for a gene panel test to produce a positive diagnosis. If this probability is at least 80% (*AI Result 1*), then the gene panel test can be directly ordered for the patient (*Action 1*); otherwise, a physician intervenes to decide between gene panel (*Action 1*) and exome sequencing (*Action 2*).

In traditional expert-only diagnostic workflows, achieving personalized diagnosis is inherently challenging. This is primarily because transition probabilities are rarely patient-specific and rely instead on generalized clinical guidelines. Even when clinicians tailor decisions based on an individual’s profile, it is difficult to quantify those probabilities without observing the process in action. In contrast, AI-based approaches can, by design, estimate these conditional probabilities in advance through probabilistic modeling. The *AI-delegation* mode was initially proposed by Gustavo et al. [24], essentially referring to a collaborative model where AI assists human experts in the clinical decision-making process. In the expert-alone mode, a decision node typically functions as a singular point where an expert selects the appropriate next action, as illustrated in Figure 2a. In contrast, the AI-delegation mode introduces a more intricate node structure, as depicted in Figure 2b. Specifically, the “decision node + N action nodes” configuration is now replaced by a sequence that begins with an AI action node, in which the AI predicts the optimal diagnostic test according to a specific patient’s phenotypes, demographics, and prior diagnostic outcomes such that the diagnostic procedure is customized to each individual patient. One or more AI result nodes subsequently follow the AI action nodes, which are partitioned by predetermined threshold values. Depending on the AI’s prediction, the process then proceeds to either an expert decision node, which mirrors the structure of the expert-alone mode, or a trivial decision node, which directly transitions to an action node without further expert input. The decision cost is thus omitted in the branch where an action node directly follows the AI result node, since test ordering is automated with no human intervention needed. The transition probabilities in AI-delegation mode therefore rely on the AI’s prediction and performance (i.e., precision; see Section 3 for an example). This expanded structure allows for more efficient AI-assisted test selection while maintaining human oversight and intervention.

## 3. Case Study: Application of DPCA to the Diagnosis Process of DD & MCA

In this section, we apply the framework proposed in Section 2 to the diagnostic procedure of *developmental delays* (***DD***) and *multiple congenital anomalies* (***MCA***).

### 3.1 Tree Representation

In accordance with the diagnostic strategies outlined by Li et al. [8], seven approaches are commonly adopted for diagnosing DD & MCA:

1. Standard testing: first-tier CMA + second-tier targeted single-gene / gene panel (GP).
2. Third-tier whole exome sequencing (ES) after standard testing above fails to provide a diagnosis.
3. First-tier CMA, second-tier ES if CMA fails to provide a diagnosis.
4. First-tier ES, second-tier CMA if ES fails to provide a diagnosis.
5. ES and CMA concurrently perform first-tier testing.
6. Third-tier genome sequencing (GS) after standard testing fails to provide a diagnosis.
7. GS alone is used for first-tier testing only.

For this case study, we simplify these seven strategies into the following four representative strategies:

1. First-tier CMA + second-tier GP;
2. First-tier CMA + second-tier GP + third-tier ES;
3. First-tier CMA + second-tier ES;
4. First-tier ES only.

**Figure 3.**
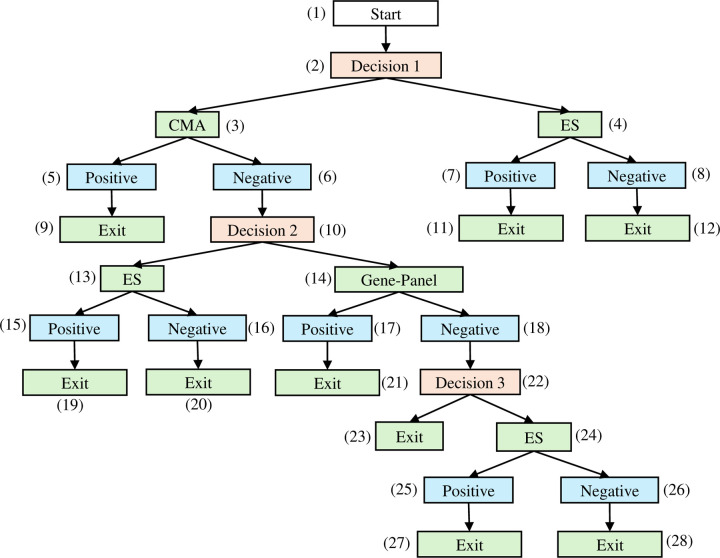
Expert-alone Mode The 4 scenarios are indicated with the following node sequences. Scenario 1 (CMA + GP): (1) – (2) – (3) – (6) – (10) – (14) – (18) – (22) – (23) Scenario 2 (CMA + GP + ES): (1) – (2) – (3) – (6) – (10) – (14) – (18) – (22) – (24) Scenario 3 (CMA + ES): (1) – (2) – (3) – (6) – (10) – (13) Scenario 4 (ES only): (1) – (2) – (4)

**Figure 4.**
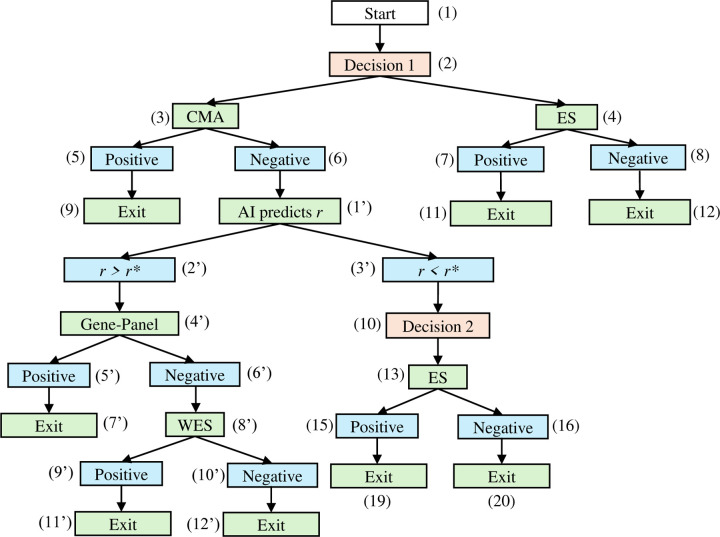
AI-delegation Mode Following the *negative* result node (6) of CMA, the *Decision 2* node (10) from the expert-alone mode is now replaced by the AI action node (1’) and two AI result nodes (2’ and 3’). The $r>r^{*}$ branch is fully AI-automated with no extra decision nodes involved. The $r<r^{*}$ branch returns the original *Decision 2* node and is assumed to be independent of AI performance. For simplicity, we assume that only ES is considered if $r<r^{*}$.

Figure 3 depicts the full diagnostic graph, where different probability assignments correspond to the four strategies listed above. Figure 4 presents a simplified workflow of the AI-delegation mode, in which the AI system assists in determining strategies (2) and (3) dynamically, i.e., whether a patient should first proceed to the GP or ES following a negative CMA result. The AI action node (1’) predicts the probability that the GP will yield a positive result. On the basis of a predetermined threshold value $r^{*}$, the model follows one of two paths: if $r\geq r^{*}$, the subtree under the AI result node (2’) is automatically activated, and the patient first undergoes GP before proceeding with ES (strategy 2); otherwise, the decision is to proceed with ES testing directly (strategy 3). The rationale behind this design is to utilize AI to examine whether the less expensive GP test is likely to succeed, thereby avoiding more costly ES.

### 3.2 Parameter Estimation

All relevant parameters are detailed in Table 1 (page 28) and grouped into the following major categories: (1) cost parameters, including the prices of individual diagnostic tests and expert consultation fees; (2) the turnaround time associated with each test and clinical consultation; (3) test performance metrics, including sensitivity, specificity, and diagnostic yield; (4) AI performance metrics, which inform the derivation of specific transition probabilities in the AI-delegation mode; and (5) parameters used to quantify the expected effectiveness. Table 2 (page 30) provides the estimation for all nontrivial transition probabilities in Figures 3 & 4 under both expert-alone and AI-delegation modes. As the total probability under each node must sum to one, we present only a representative portion of the distribution. The next few subsections elaborate on how the nontrivial probabilities in Figures 3 & 4 are estimated.

#### 3.2.1 Expert-alone Scenarios

In real-world clinical settings that rely solely on human expertise, a standardized and institution-wide diagnostic protocol is often applied uniformly across all patients. Under such predefined protocols, the transition probability from a decision node to an action node is deterministic, taking values of either 0 or 1. For example, at the *Decision 1* node in Figure 3, if the institutional guideline mandates the CMA as the first-tier test, the probability of selecting the CMA is $P_{2,3}=1$. All action nodes represent diagnostic tests that yield either a positive or negative result; the probability associated with a positive result is therefore equivalent to the diagnostic yield of the respective test, which can be estimated as the proportion of positive outcomes relative to the total number of tests administered (e.g., $P_{3,5}=P\left(Y_{0}=1\right)=YD_{0}$).

Personalized diagnosis would tailor these transition probabilities based on individual patient characteristics, such as phenotypic features, demographics, or clinical presentation, rather than adhering to a fixed diagnostic sequence. In such settings, $P_{ij}$ becomes a conditional probability that can be estimated from patient-specific factors and historical clinical data. Estimation may be performed through simple empirical ratios (e.g., the proportion of patients with DD and MCA phenotypes who undergo CMA as the first-tier test) or through more sophisticated predictive models based on patient-level covariates, which can be better achieved through the integration of AI-based models introduced in Section 3.2.2.

However, owing to the multilayered structure of diagnostic trees, accurately estimating conditional probabilities at deeper nodes becomes increasingly impractical. For example, deriving a probability such as $P_{24,25}=P\left(YD_{2}=1|patient,YD_{0}=0,YD_{1}=0\right)$ may require extensive data that are rarely available in clinical practice. To address this limitation, conditional probabilities at deeper layers can be approximated via marginal probabilities as a practical solution. When constructing personalized diagnostic pathways, it is thus essential to balance the trade-off between predictive accuracy and computational feasibility when deciding whether to adopt marginal or conditional probability estimation.

#### 3.2.2 AI-delegation branch

The AI-delegation branch introduces two distinct probability types not presented in the expert-alone mode: the probability associated with the AI’s result node $\left(P_{1^{\prime },2^{\prime }}\right)$ and the GP’s diagnostic yield conditioned on the AI’s prediction $\left(P_{14^{\prime },17^{\prime }}\right)$. When the prediction score, r, varies at the population level, the corresponding probability $P_{1^{\prime },2^{\prime }}=P\left(r\geq r^{*}\right)$ lies within the range of (0,1). However, when the prediction is conditioned on a specific patient, i.e., $P_{1^{\prime },2^{\prime }}=P\left(r\geq r^{*}|patient\right)$, the prediction result r becomes a fixed value on the basis of the patient’s input data and the AI model; thus, $P_{1^{\prime },2^{\prime }}=0$, *or* 1 depends on the threshold value $r^{*}$. Furthermore, the diagnostic yield of the GP $\left(P_{14^{\prime },17^{\prime }}\right)$ under the AI-delegated branch $r\geq r^{*}$ differs from that in the expert-alone mode. Rather, it reflects AI performance and can be formally defined as $P\left(y_{1}=1|r>r^{*}\right)=\frac{P\left(True\;Positive\right)}{P\left(Predicted\;Positive\right)}$ (i.e., AI precision). Assuming that the AI model is unbiased, its predictive performance should be treated as independent of specific patient phenotypes or demographics. The subsequent nodes under the $r<r^{*}$ branch revert to expert judgment and remain unaffected by the AI’s performance; thus, the transition probabilities follow the same estimation as in the expert-alone mode.

### 3.3 Expected effectiveness

Recall the *outcome state* defined in Section 2.3. In this case study, all diagnostic tests considered produce dichotomous outcomes, leading to either positive or negative diagnostic results as the final outcome (i.e., $O_{i}=1\;or\;0$). In the context of this example, the EGU of an outcome can thus be defined as the sum of the expected utility of a true positive result $\left(U_{TP}\right)$ and the *disutility* of a false positive result $\left(U_{FP}\right)$, mathematically expressed as

$$EGU_{i}=PPV_{i}*U_{TP}\left(1-PPV\right)*U_{FP}\;\text{for}\;O_{i}=1,$$

Similarly, the EGU of a negative outcome is given by

$$EGU_{i}=NPV_{i}*U_{TN}\left(1-NPV\right)*U_{FN}\;\text{for}\;O_{i}=0.$$

*The* positive predictive value (PPV) and negative predictive value (NPV) reflect the accuracy of the final diagnostic results, which can be derived from the disease prevalence $\left(p\right)$ as well as the sensitivity $\left(Se\right)$ and specificity $\left(Sp\right)$ of the conducted diagnostic tests to yield the final results (Table 1) [25]:

$$PPV=\frac{Se*p}{Se*p+\left(1-Sp\right)*\left(1-p\right)};$$

$$NPV=\frac{Se*\left(1-p\right)}{Sp*\left(1-p\right)+\left(1-Se\right)p}.$$

Since each diagnostic strategy outlined in Section 3.1 leads to multiple possible outcomes, the expected effectiveness is the probability-weighted sum of the utilities across all outcome states. The decay rate parameter for the TAT, λ, is restricted to values between 0.01 and 0.1 to ensure a gradual and steady decline in utility with increasing diagnostic delays, which is appropriate for the clinical context of DD & MCA.

### 3.4 Cost-effectiveness Analysis Demonstration

To illustrate how PRICE can be practically implemented, we present a demonstration through an interactive web-based dashboard ^1^. This dashboard enables real-time evaluation of diagnostic strategies under varying clinical conditions and individual preferences by allowing users to adjust key parameters such as test costs, test performances, and utility weights. We provide several examples below to illustrate how changes in parameters affect cost-effectiveness and how this information can assist with patient decision-making.

#### 3.3.2 Example 1

Consider a hypothetical case of a 2-year-old patient presenting with DD & MCA phenotypes, and four health institutions that each implement one of the expert-alone diagnostic strategies described in Section 3.1 as their standard clinical protocol. To obtain a preliminary estimate of the potential costs prior to the diagnostic process, the patient’s caregiver can input specific parameter values to simulate cost-effectiveness outcomes for all scenarios.

**Figure 5.**
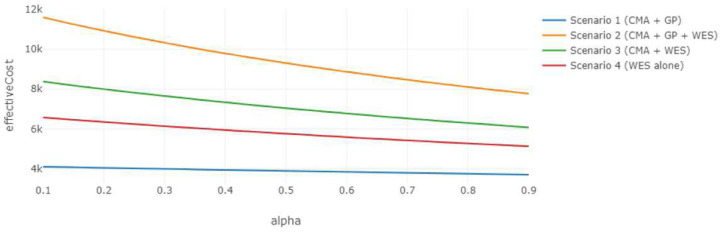
Effective Cost vs. Alpha, Expert-alone Scenarios Effective cost of expert-alone diagnostic strategies across varying values of the weighting parameter α (i.e., the relative importance of diagnostic accuracy placed by the patient), demonstrating the per effective unit cost. The parameter values are taken from the reference distributions and fixed values in Table 1. NPVs and PPVs are designed to be entered in the dashboard and can be estimated with the prevalence, sensitivity and specificity values provided. $C_{E}=165$, $C_{0}=825$, $C_{1}=1500$, $C_{2}=4589.4$, $YD_{0}=0.1$, $YD_{1}=0.11$, $YD_{2}^{1}=0.37$, $YD_{2}^{3}=0.33$, λ=0.03, $U_{TP}=1$, $U_{FP}=-1$, $U_{TN}=1$, $U_{FN}=-1$.

**Figure 6.**
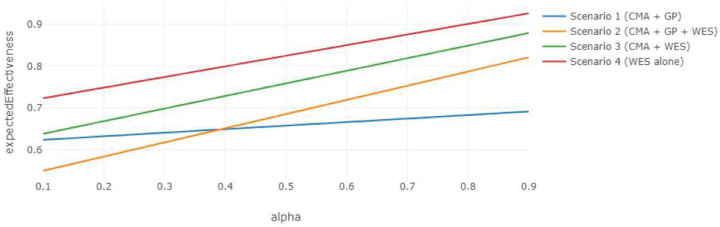
Expected Effectiveness vs. Alpha, Expert-alone Scenarios Expected effectiveness of expert-alone diagnostic strategies across varying values of the weighting parameter α, demonstrating the overall clinical effectiveness. The parameter values are the same as above.

Figure 5 illustrates how the effective cost of each strategy varies with changes in the weighting parameter, α, which controls the relative importance of diagnostic performance versus turnaround time. The CMA + GP pathway in scenario 1 emerges as the most cost-efficient strategy, followed by the first-tier ES of scenario 4. However, the expected effectiveness outcomes displayed in Figure 6 demonstrate that ES as a first-tier test yields markedly higher expected effectiveness than CMA + GP does, particularly when the diagnostic performance is prioritized (i.e., higher values of α). This reflects a fundamental trade-off between per effective unit cost (i.e., value of money) and overall clinical effectiveness. If the caregiver prioritizes diagnostic accuracy and the potential for early therapeutic intervention, first-tier ES may be the preferred strategy even if its effective cost is relatively higher.

#### 3.3.3 Example 2

**Figure 7.**
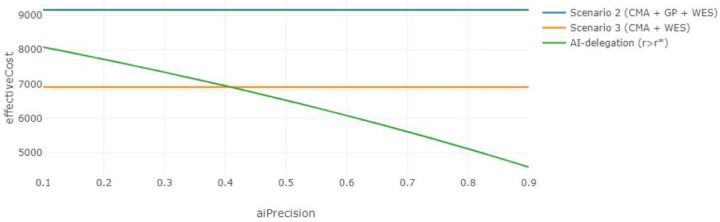
Effective Cost vs. AI Precision The effective cost of the AI-delegation mode compared to those of scenarios 2 and 3 under the expert-alone mode across varying values of AI precision. The weighting parameter α is set to 0.5, and the other parameters take the same values as above.

**Figure 8.**
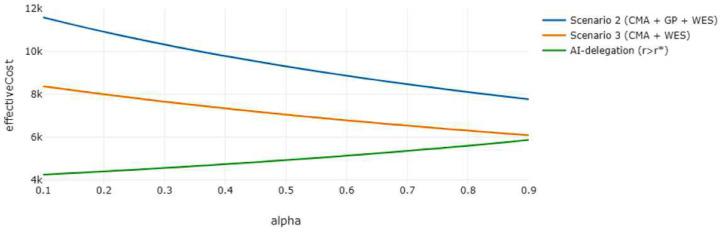
Effective Cost vs. Alpha, AI-delegation Effective cost of the AI-delegation mode compared to scenarios 2 and 3 under the expert-alone mode across varying values of the weighting parameter α. AI precision is set to 0.87, and the other parameters take the same values as above.

Now consider an alternative situation in which another healthcare institution employs an AI-assisted diagnostic model, corresponding to the AI-delegation mode where the CMA + GP + ES pathway in scenario 2 is partially automated if $r\geq r^{*}$. If the AI’s prediction score falls below the threshold value $\left(r<r^{*}\right)$, then the pathway defaults to expert-alone scenario 3, where the patient transitions from the CMA to the ES. As illustrated in Figure 7, the effective cost of the AI-automated pathway decreases significantly as the AI’s predictive precision increases, eventually outperforming both expert-alone scenarios 2 and 3 once the precision exceeds 0.43. This pattern is further supported in Figure 8, which sets the AI precision at 0.87 [26], confirming the superior cost-effectiveness of the AI-delegated approach under high predictive performance. The implementation of such high-precision AI models provides a more cost-effective and personalized alternative to standard protocols. From a decision-making perspective, this configuration offers an appealing middle ground if the caregiver wishes for ES to be included in the diagnostic process but prefers to defer its use until necessary.

## 4. Discussion

We introduce a dynamic, recursive cost estimation framework for rare disease diagnosis, integrating AI-delegation with expert decision-making. Unlike traditional static models, our approach captures the sequential dependencies inherent in diagnostic processes, reflecting how early-stage clinical decisions influence downstream costs. To facilitate practical applications, we developed a web-based tool (link) enabling users to construct custom diagnostic trees. This interactive platform supports real-time cost estimation via the *BECC* algorithm, allowing dynamic editing of nodes and parameters. This tool empowers clinicians and patients to visualize and compare the expenses of various diagnostic pathways effectively. To complement the cost analysis, we also propose a utility-based assessment to evaluate the expected effectiveness of diagnostic strategies, which aligns with the broader concept of health-related quality of life (HRQL), a fundamental measure of healthcare intervention benefits [27]. Recognizing that utility values are subjective and vary across individuals, our framework allows for a standard, yet personalized utility assessment, ensuring that evaluations reflect an individual patient’s context and preference.

In clinical cost-effectiveness analysis, several structural approaches are commonly utilized to evaluate the outcomes of medical interventions, with the exception of the decision model employed in our study. Among these, the Markov model is commonly adopted to simulate transitions between discrete health states over time, capturing the progression of chronic diseases and the impact of different treatments [28]. For example, Townsend et al. [29] applied a Markov-based model to simulate hepatitis C across six health states, enabling the comparison of lifetime costs and health outcomes associated with various treatment strategies. The model integrates transition probabilities, costs, and utility values to assess cost-effectiveness under different clinical scenarios. In a more dynamic application, Bennett & Hauser [30] developed an AI framework that combines Markov decision processes and dynamic decision networks to simulate clinical decision-making. This framework learns from electronic health records to develop complex treatment plans by updating belief states about patient health status. Our study adopts a decision tree model, which is suitable for evaluating short-term interventions and diagnostic procedures with a limited number of discrete outcomes. With a focus on the diagnostic process, our framework introduces two key innovations. First, it offers a dynamic and adaptive structure that accommodates modifications in the diagnostic process, including scenarios where AI is incorporated to assist in test selection and decision-making. Once the diagnostic tree structure is defined, this cost-effectiveness analysis framework can be applied consistently across any scenario—whether AI-assisted or expert-alone. Second, we propose a new method for assessing the effectiveness of a diagnostic process by integrating both diagnostic utility and turnaround time into a composite effectiveness metric, allowing for straightforward comparisons among multiple diagnostic strategies.

Given the increasing emphasis on personalized diagnostics, it is essential to consider the evolving role of human-AI collaboration in clinical decision-making. Recent studies have demonstrated that integrating AI systems with clinician expertise can increase diagnostic accuracy and efficiency. For example, Lee et al. [31] developed a machine learning–based decision support system for stroke rehabilitation assessment, which automatically generated patient-specific analyses for therapists and refined outputs on the basis of expert feedback. This mutual learning process between AI and clinicians facilitated complex decision-making with improved accuracy. Similarly, Frazer et al. [32] investigated four human–AI collaboration scenarios in mammographic screening: AI single-reader, AI reader replacement, AI bandpass, and AI triage. These configurations demonstrated that collaborative approaches could enhance diagnostic performance while ensuring that human expertise remains central, particularly in complex or uncertain cases. Recognizing the growing potential and adoption of human-AI collaboration in healthcare, our framework is designed with the flexibility to incorporate AI-delegation modes for cost analysis.

A critical consideration for implementing the PRICE framework in real-world clinical settings is the challenge of obtaining reliable and comprehensive data to inform model parameters. Estimating transition probabilities between nodes, such as test selection probabilities or diagnostic yields, requires access to detailed, longitudinal patient records, which are often incomplete or inconsistently documented [33, 34]. Furthermore, patient-level covariates (e.g., demographic factors, clinical history, etc.) needed for personalized probability estimation may be unavailable or non-standardized across healthcare systems [35, 36]. Other parameters, such as different costs and turnaround times, may also pose additional difficulties, as they vary widely across institutions, geographic regions, and payer systems [37]. These discrepancies can lead to substantial heterogeneity in parameter values. Addressing these challenges will require collaborative efforts to standardize diagnostic data collection, make transparent information sharing, and integrate real-time data from electronic health records into decision-analytic frameworks.

Another essential factor in making this conceptualized workflow truly actionable for individual patients is cost estimation. However, in practice, healthcare costs remain highly opaque due to the involvement of third-party payers (i.e., insurance companies). These payers may use such frameworks primarily to minimize overall expenditures, often without adequately considering clinical effectiveness. This dynamic likely contributes to the frequent denial of claims for clinically validated exome sequencing tests by insurers. Enhancing cost transparency and involving patients in shared decision-making, especially when financial implications may directly affect their daily socioeconomic lives, is both important and necessary. Without clear visibility into costs, even the most effective AI-driven tools or clinical tests may fail to reach patients, particularly those from economically disadvantaged backgrounds.

## 5. Conclusion

By modeling diagnostic pathways as recursive decision-action-result trees, the PRICE framework captures the dynamic nature of clinical decision-making and supports personalized cost estimation. Its ability to integrate AI components into the analysis enables more nuanced modeling of modern diagnostic workflows where AI assists in decision making. The framework also incorporates a utility-based model that accounts for diagnostic timeliness and outcome quality, providing a more comprehensive evaluation of effectiveness. An interactive tool further allows real-time scenario analysis tailored to patient-specific and institutional contexts. To ensure equitable implementation, future efforts should focus on improving cost transparency and actively involving patients in the decision-making process.

## Figures and Tables

**Table 1.** Parameters and notations used in framing the PRICE model and their estimations in the case study.

Parameter Category	Notation^1^	Description	Reference Values
Cost	$C_{E}$	Expert fee	165 [8]
	$C_{0}$	Cost of CMA test	Gamma (2010, 2.44) [8]
	$C_{1}$	Cost of GP test	Ranges from $1450 to $1750 [38]
	$C_{2}$	Cost of ES test	Normal (4589.4, 45) [8]
Turnaround Time	$T_{0}$	CMA turnaround time	2 weeks [39, 40]
	$T_{1}$	GP turnaround time	4 weeks [41, 42]
	$T_{2}$	ES turnaround time	8 weeks [43]
	$T_{E}$	Physician consultancy	4 weeks ^2^
	$T_{AI}$	AI decision-making	0 weeks
Test Result	$Y_{0}$	Test result of CMA	NA
	$Y_{1}$	Test result of gene-panel	NA
	$Y_{2}$	Test result of WES	NA
Test Performance	$YD_{0}$	CMA diagnostic yield	Beta (154, 1382) [8]
	$YD_{1}$	GP diagnostic yield	Beta (24, 89) [8]
	$YD_{2}^{1}$	1^st^ tier ES diagnostic yield	Beta (27, 46) [8]
	$YD_{2}^{2}$	2^nd^ tier ES diagnostic yield	0.35 [8]
	$YD_{2}^{3}$	3^rd^ tier ES diagnostic yield	Beta (228, 464) [8]
	$Se_{0}$	CMA sensitivity ^3^	0.9068 [44]
	$Sp_{0}$	CMA specificity	0.9440 [44]
	$Se_{1}$	GP sensitivity	0.8960 [45–47] ^4^
	$Sp_{1}$	GP specificity	0.9250 [26–28] ^5^
	$Se_{2}$	ES sensitivity	0.9593 [48, 49] ^6^
	$Sp_{2}$	ES specificity	0.9933 [33, 34] ^7^
AI Performance	r	Likelihood of a positive result from GP test	NA
	$P^{AI}$	AI’s precision	0.87 [26]
Disease Prevalence	p	DD & MCA prevalence	0.1665 [50]
Effectiveness	α	Weighting parameter	Ranges from 0 to 1
	λ	Tuning parameter	Ranges from 0.01 to 0.1
	$U_{TP}$	Utility of a true positive test result	Ranges from 0 to 1
	$U_{FP}$	Utility of a false positive test result	Ranges from −1 to 0
	$U_{TN}$	Utility of a true negative test result	Ranges from 0 to 1
	$U_{FN}$	Utility of a false negative test result	Ranges from −1 to 0

1 All the notations’ subscripts in Table 1 are irrelevant to the node numbers shown in Figure 3 & 4. For simplicity, subscripts 0, 1, and 2 denote CMA, GP and ES, respectively.

2 A rough estimation based on the expert’s opinion, can vary by different factors (e.g., patients’ urgency).

3 Sensitivity (specificity) refers to a test’s ability to correctly identify individuals who truly (does not) have the disease and is quantified as the proportion of true positives (negatives) among all positive (negative) cases [51]. A test may exhibit high sensitivity yet still produce a low diagnostic yield if the disease prevalence in the tested population is low.

4–7 These reference values are the average values taken from multiple sources.

Note: Table 1 can be placed after Section 3.2.

**Table 2:** Estimation of $P_{ij}$ under various modes.

Edge	Mode	Formulation	Explanation
$P_{2,3}$	Expert-alone & AI-delegation	1 *or* 0	Assume each institution follows a standard protocol.
$P_{3,5}$	Expert-alone & AI-delegation	$P\left(Y_{0}=1\right)YD_{0}$	Diagnostic yield of CMA.
$P_{4,7}$	Expert-alone & AI-delegation	$P\left(Y_{2}=1\right)YD_{2}^{1}$	Diagnostic yield of a first-tier WES.
$P_{10,13}$	Expert-alone & AI-delegation	1 *or* 0 for expert-alone, 1 for AI-delegation	Assume each institution follows a standard protocol.
$P_{13,15}$	Expert-alone & AI-delegation	$P\left(Y_{2}=1|Y_{0}=0\right)≅YD_{2}^{2}$	The yield of WES given a negative result from the previous CMA test. Can be directly approximated with the marginal yield of a second-tier WES.
$P_{14,17}$	Expert-alone	$P\left(Y_{1}=1|Y_{0}=0\right)≅YD_{1}$	The yield of gene-panel given a negative result from the previous CMA test. Can be directly approximated with the marginal yield of gene-panel.
$P_{22,23}$	Expert-alone	1 *or* 0	Assume each institution follows a standard protocol.
$P_{24,25}$	Expert-alone	$P\left(Y_{2}=1|Y_{1}=0,Y_{0}=0\right)≅YD_{2}^{3}$	The yield of WES given a negative result from both CMA and gene-panel in the previous tiers of test. Can be directly approximated with the marginal yield of a third-tier WES.
$P_{1^{\prime },2^{\prime }}$	AI-delegation	$P\left(r>r^{*}\right)=1\;or\;0$	$r^{*}$ is always a fixed value for a given patient, thus $P\left(r>r^{*}\right)$ is binary (either 1 or 0).
$P_{4^{\prime },5^{\prime }}$	AI-delegation	$P\left(Y_{1}=1|r>r^{*}\right)=P^{AI}$	$\begin{array}{l} P\left(y_{1}=1|r>r^{*}\right)\\ =\frac{P\left(y_{1}=1|r>r^{*}\right)}{P\left(r>r^{*}\right)}\\ =\frac{P\left(TP\right)}{P\left(\text{Predicted}\;\text{positive}\right)}=P^{AI} \end{array}$
$P_{8^{\prime },9^{\prime }}$	AI-delegation	$P\left(Y_{2}=1|r>r^{*},Y_{1}=0,Y_{0}=0\right)≅YD_{2}^{3}$	Since ES yield is irrelevant to AI’s prediction, this can still be estimated with the marginal yield of a third-tier WES.

Note: Table 2 can be placed after Section 3.2, following Table 1.

## Data Availability

The source codes of the web-based tools developed for this study are available at [DD-MCA-Diagnosis-Process-Cost-Model] and [PRICE-React-D3-Tree] repositories. The web-based tool for constructing diagnostic trees is published at http://165.22.13.117:4833/. No additional research data involved.

## Footnotes

1 For demo, please follow the instructions at [DD-MCA-Diagnosis-Process-Cost-Model].
